# Stakeholder Analysis of the Waste Electrical and Electronic Equipment Internet Recycling Industry

**DOI:** 10.3390/ijerph191610003

**Published:** 2022-08-13

**Authors:** Tingting Liu, Qian Zhang, Zichen Zheng, Shangyun Wu, Zhixiong Weng

**Affiliations:** Institute of Circular Economy, Beijing University of Technology, Beijing 100124, China

**Keywords:** waste electrical and electronic equipment, internet recycling, stakeholder analysis, China

## Abstract

With the acceleration of the digitization process and the popularization of the internet, the recycling of waste from electrical and electronic equipment (WEEE) has become a potential and emerging recycling method. Stakeholders in the WEEE internet recycling industry have different roles which need to be clarified. The stakeholder structure and relationships, and stakeholder characteristics, lie at the core of recycling system governance. Therefore, it is necessary to identify stakeholders in the existing WEEE Internet recycling industry. This study selected 10 important stakeholders and classified them into key, potential, and marginal stakeholders using the Mitchell scale while analyzing their characteristics and interactions. The results showed that internet recycling companies, government, residents, and traditional recyclers are key stakeholders that are important for promoting the development of the industry. Based on the above analysis, policy advice is proposed to provide directions for the improvement of the WEEE industry.

## 1. Introduction

The production and consumption of electrical and electronic equipment (EEE) is increasing yearly, leading to increasing waste EEE (WEEE) [1]. WEEE is one of the fastest-growing solid wastes in the world. The global annual growth rate of WEEE is estimated to be ca. 4% [2,3]. According to the “China White Paper on Waste Electrical and Electronic Equipment Recycling Industry of 2021” released by the China Household Electrical Appliances Research Institute in May 2022, the theoretical scrap rate of WEEE in 2021 increased by 12.6% over 2020 [4]. China’s WEEE is showing a rapid growth trend. The recycling, treatment, and reuse of the mass-produced WEEE in a safe, environment-friendly, and efficient manner has become an urgent problem to be solved.

With the management of WEEE becoming an increasingly crucial concern, new models of Internet recycling have emerged in the field of WEEE recycling. Various scholars have researched WEEE management. For example, Liu et al. [5] studied the competition between the formal and informal sectors and constructed a game model of the dismantling and refurbishment process, thereby identifying the optimal recycling fee levied on the producer and the corresponding subsidies to the formal sector. Lu et al. conducted a comparison of WEEE reuse policies and practices in China and the European Union (EU) and revealed that China’s policies need to be more systematized like the EU’s; moreover, they proposed further research on the drivers and barriers to reuse to support more effective and efficient WEEE management [6]. However, due to its broad development prospects, further research is necessary on Internet recycling. This is especially important in developing countries. Developed countries have established relatively standardized management systems for the recycling of renewable resources, while recycling systems in developing countries need to be improved. The effectiveness of the Internet recycling model in the recycling of household renewable resources in developed countries is far less than that of China [7]. Therefore, more researchers are shifting their focus to the WEEE internet recycling industry. Sun et al. demonstrated the advantages of the “Internet + recycling” platform [8]. Jian et al. [9] used game models to discuss the collaborative strategy in the development of the “Internet + Recycling” platform. Zuo et al. [10] studied the O2O model of WEEE recycling in China, and Xue et al. [11] studied the intelligent collection mode of urban resources in China and found huge development potential.

In recent years, in China, the “Internet +” recycling model has continued to develop. In general, we can summarize it into three categories. The first mode is the recycling mode based on big data. This model is a recycling system based on big data constructed by WEEE recycling companies in order to improve operational efficiency and develop digital technologies. The second mode is the multi- stakeholder cooperative recycling mode. The WEEE recycling industry chain involves multiple stakeholders. Under the background of “Internet +” recycling, some companies have developed a multi-stakeholder cooperative recycling system through cooperation. The third mode is the recycling mode combined with garbage classification. This model combines Internet recycling and garbage classification, and adopts methods such as setting up smart recycling bins and making appointments to pick up items, and incorporates WEEE into the domestic waste classification network for recycling.

In order to gain a deeper understanding of how the WEEE recycling industry in China works, we need to discuss further the stakeholders within the industry. In fact, with increasing WEEE management research, people are gradually realizing that each stakeholder has a different role in the WEEE recycling industry, and this influences the effect of the industry’s chain policy [12]. Analyzing the roles of different stakeholders in the WEEE recycling industry can help us better understand how the WEEE recycling industry operates and allow us to manage it better. A multi-criteria decision-making method has been developed by integrating the Interval Analytic Hierarchy Process and the Interval VIKOR method for China’s stakeholders to select the most efficient portfolio for solving the severe problems caused by informal disposal of WEEE [13]. Tasaki et al. [14] surveyed stakeholders’ perceptions of the concept of extended producer responsibility (EPR), including its aims, application, and rationale, and demonstrated the diversity in stakeholders’ perceptions. Moreover, they identified several patterns relating stakeholders’ perceptions and attributes. One study surveyed over 400 respondents from EPR stakeholders worldwide to ascertain understanding and views on the purpose of EPR internationally [15]. This reflects an increasing recognition of how the characteristics of stakeholders influence decision- and policy-making processes.

The WEEE recycling industry requires essential stakeholders involved in implementing circular economy approaches to work together. However, although many previous studies have considered this from the perspective of stakeholders, they have different descriptions and definitions of the stakeholders in the WEEE recycling industry, and few studies have considered all stakeholders. This makes our work more subjective in finding research topics and identifying stakeholders, making it difficult to get a systematic and comprehensive view of the industry. Considering this, we aimed to comprehensively study the emerging industry of WEEE Internet recycling, by analyzing its existing stakeholders to further identify the ones that play a vital role in the industry. This can help us find the focus of research more easily in future work. Currently, the most widely used stakeholder classification methods are the multi-dimensional classification method and the Mitchell score method. The latter method divides the industry into three types of stakeholders, which can help us accurately distinguish the importance of stakeholders in the WEEE Internet recycling industry. Therefore, we hope to improve the Mitchell score method based on the characteristics of the WEEE Internet recycling industry. Through this approach, we divided the WEEE internet recycling industry into key, potential and marginal stakeholders. The characteristics and interaction among the stakeholders were investigated to promote the sustainable development of the WEEE recycling industry and provide a theoretical basis for the government to supervise and manage the industry and provide companies with operational decision-making references. Furthermore, it also contributes to environmental protection by promoting the construction of green supply chains.

## 2. Methods

### 2.1. Determination of Stakeholder Scope

Several studies have investigated the WEEE recycling system, and researchers have differing definitions of stakeholders (Table 1). We compared the findings of existing studies, providing a starting point for determining the scope of our subsequent stakeholder analysis.

Based on stakeholder theory, published literature, and analysis of the development status of the WEEE Internet recycling industry, we selected 15 stakeholders. These included residents, electronic product manufacturers, electronic retailers, Internet recycling companies, traditional recyclers, scavengers, disassembly and treatment companies, reusing companies, waste treatment companies, hazardous waste treatment companies, garbage treatment companies, individual repair stations, logistics companies, government and non-governmental organizations, and third party payment platforms. These were narrowed down through expert scoring to 10 stakeholders of the WEEE Internet recycling industry (Table 2).

### 2.2. Classification Method of Stakeholders

#### 2.2.1. Classification Dimensions of Stakeholders

Various methods exist for classifying stakeholders, including generalized stakeholder taxonomy and multidimensional classifications. However, Mitchell and Wood proposed a clear and easy-to-implement scoring method to classify stakeholders [23]. According to the Mitchell scoring method, the core of stakeholder theory lies in the confirmation of stakeholders and the analysis of their characteristics, which determine who the stakeholders are and their importance. On this basis, Mitchell scored stakeholders according to three dimensions: legitimacy, power, and urgency. The higher the score, the more closely related to the interests of the company. Our work improves on Mitchell’s foundation, by changing legitimacy to initiative. The reason for this change is that we think that the WEEE Internet recycling industry is an emerging industry, especially in China. Its laws and related management systems are not fully established, and the degree of dependence of these stakeholders on the industry is more reflected in their own initiative. The stakeholders in the WEEE Internet recycling industry were subdivided according to the following three dimensions: initiative, importance, and urgency. The 18 questionnaires were issued to relevant experts in the industry (e.g., experts from universities, institutes, governments, and enterprises) and household electrical industry Association and the Recycling Association. The experts were asked to score the 10 stakeholders on three dimensions (initiative, importance, and urgency) and fill in the evaluation form of stakeholder experts for WEEE Internet recycling. The specific dividing dimensions and scoring rules were as follows:

(1) The initiative dimension refers to the immediacy of a stakeholder’s demand for the WEEE Internet recycling industry, that is, the stakeholder has a direct interest demand in WEEE Internet recycling. The initiative of the 10 listed stakeholders was assigned a score of 1–5. The higher the initiative value of a stakeholder, the higher the urgency for Internet recycling.
(2) The importance dimension is derived from the power dimension in the Mitchell scoring method and refers to the degree to which the status, ability, and corresponding behavior of a related interest group have an impact on the WEEE Internet recycling industry. Moreover, it will also be directly affected by the trend of the WEEE Internet recycling industry. The importance of the 10 listed stakeholders was assigned a score of 1–5. The more importance a stakeholder is assigned, the more influence its status, capabilities, and corresponding behaviors will have in the industry.
(3) The urgency dimension refers to whether the needs of a certain stakeholder group can immediately attract the attention of the WEEE Internet recycling industry. The absence of this stakeholder will lead to the normal operation of the whole industry. The urgency of the 10 listed stakeholders was assigned a score of 1–5. The higher the urgency assigned to a stakeholder, the more immediate attention will be paid to its needs.

#### 2.2.2. Classification Principle of Stakeholders

Based on the Mitchell classification method, existing research, and the results of our expert consultation questionnaire, stakeholders in WEEE Internet recycling were divided into three levels: key, potential, and marginal stakeholders. Key stakeholders reflect those stakeholders that scored > 4 points in two of the three dimensions; Potential stakeholders reflect those stakeholders that scored 3–4 points in at least two dimensions; Marginal stakeholders reflect those that scored < 3 points in at least two dimensions (Table 3).

## 3. Results

### 3.1. Classification Results

#### 3.1.1. Dimension Scores

According to the results of the questionnaire, the initiative scores are shown in Table 4. The initiative dimension analysis showed that the highest scoring stakeholder was Internet recycling companies, scoring 4.88 points. The initiatives of governments, residents, and traditional recyclers were high also, with all scoring > 3 points. The initiative scores of electronic products manufacturers, disassembly and treatment companies, reusing companies, hazardous waste treatment companies, non-government organizations, and garbage treatment companies were low, scoring < 3.

The importance dimension analysis showed that the importance of Internet recycling companies, governments, residents, and traditional recyclers was relatively high, scoring > 4 points. The importance scores of electronic product manufacturers and disassembly and treatment companies were lower, scoring between 3 and 4 points. The importance of hazardous waste treatment companies and non-government organizations were the lowest, scoring < 3 points (Table 5).

The urgency dimension analysis showed that Internet recycling companies, governments, residents, and traditional recyclers had the same degree of urgency and importance, scoring > 4 points. Electronic product manufacturers and disassembly and treatment companies had a lower degree of urgency, scoring 3 to 4 points. Reusing companies, hazardous waste treatment companies, non-governmental organizations, and garbage treatment companies had the lowest degree of urgency, scoring < 3 points (as shown in Table 6).

#### 3.1.2. Three-Dimensional Classification Results

Table 7 shows the three-stage numerical classification results for the 10 types of stakeholders.

**Figure 1:**
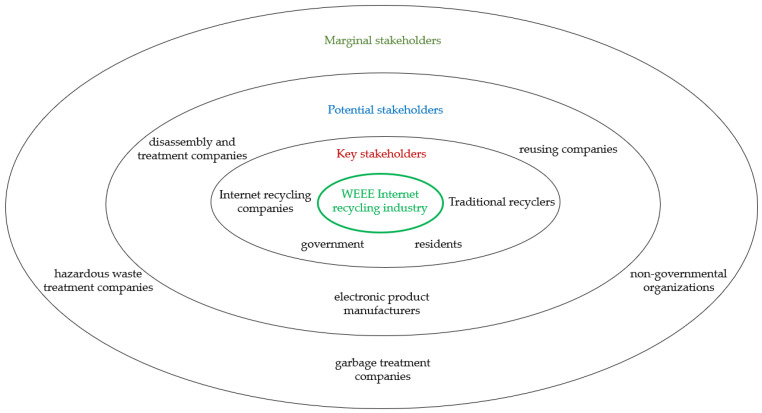
WEEE internet recycling industry stakeholder hierarchy.

According to the scores of three dimensions of the WEEE Internet recycling industry stakeholders, the stakeholders can be divided into key stakeholders, potential stakeholders, and marginal stakeholders (Figure 1).

Key stakeholders in WEEE Internet recycling are internet recycling companies, governments, residents, and traditional recyclers. These stakeholders are crucial to the development of Internet recycling and directly affect the survival of the industry.

Potential stakeholders in WEEE Internet recycling include disassembly and treatment companies, reusing companies, and electronic product manufacturers. They have a close interest relationship with the development of Internet recycling and their interest requirements are usually paid attention to and satisfied. Generally, their interest demands are not as strong as those of key stakeholders. Once their interest demands are ignored or not satisfied, their reaction is strong, which may affect the sustainable development of Internet recycling.

Marginal stakeholders in WEEE Internet recycling include hazardous waste treatment companies, non-governmental organizations, and garbage treatment companies. They are passively affected by the development of Internet recycling. They only play an auxiliary role in the development of Internet recycling but cannot directly affect it. They have the lowest impact on the development of Internet recycling.

### 3.2. Behavioral Characteristics of Key Stakeholders

Internet recycling companies and traditional recyclers are two direct recycling subjects that have a direct interest relationship with other stakeholders. As a key stakeholder in the development of the industry, the government guides, supervises, and encourages recycling companies, and has a direct interest relationship with recycling companies. Residents are an important group to determine the efficiency of WEEE recycling and disposal and are the first link in the whole recycling chain. These four key stakeholders will have a significant impact on the development of WEEE Internet recycling. Based on this background, this section will analyze the behavioral characteristics of these four key stakeholders: Internet recycling companies, governments, residents, and traditional recyclers.

#### 3.2.1. Internet Recycling Companies

Internet recycling companies are the main recycling subjects of WEEE Internet recycling. Internet recycling has increased in popularity as a recycling model in recent years. The model combines offline and online approaches to realize a network platform that orders scrap materials via a modern logistics system, which eliminates the limits of space and time between residents and recycling companies. Residents can recycle waste using their smartphones, which partly motivates their willingness and enthusiasm for recycling. Internet recycling companies can integrate online and offline information and reduce information asymmetry. Modern logistics save on the transportation and transaction costs of recycling. The combination of online and offline information saves residents’ time cost and improves their convenience, thus improving the recycling and utilization rate of WEEE.

#### 3.2.2. Government

The government is the direct supervisor and manager of WEEE Internet recycling. The main bodies that the government can act on include producers, recyclers, processors, and consumers. The government adopts the mode of multi-department coordinated management to promote WEEE recycling.

#### 3.2.3. Residents

Residents are the main consumers of electrical and electronic products. Residents have the characteristics of wide distribution and small individual differences. The electrical and electronic products mainly include TVs, refrigerators, washing machines, air conditioners, computers, mobile phones, and some small digital products. Residents are the main source of WEEE and are relatively dispersed. Therefore, the participation degree of residents in WEEE Internet recycling is very important to improve the recovery rate. Residents can dispose of WEEE in the following ways: sell it to traditional recyclers in the community, make an appointment for recycling using an Internet recycling APP, trade in the old products for new ones or leave them at home. Residents’ specific choice of recycling methods will mainly be measured by the economic benefits, convenience, and safety of various recycling methods. Residents tend to choose channels with a high price, high convenience, and good security for recycling. Especially for computers, mobile phones, and other electronic products, consumers pay more attention to the safety of recycling.

#### 3.2.4. Traditional Recyclers

Traditional recyclers mainly include individual recyclers, most of whom adopt the family-style recycling mode. Tricycles are used as their means of transportation to carry out recycling from street to street, or fixed booths are selected in the community for consumers to come to the door for consultation. Individual recyclers, who evade store rents and do not pay value-added tax or income tax, fall into the category of informal recyclers. At present, most WEEE, especially the waste electrical appliances, is still mainly recycled by individual waste recyclers with better liquidity. Urban areas are often the main activities of individual recyclers.

### 3.3. Relationship among Key Stakeholders

Through the identification and division of the above stakeholders, the government, residents, Internet recycling companies, and traditional recyclers were identified as the key stakeholders of the WEEE Internet recycling industry. These four types of key stakeholders are the main subjects of the WEEE Internet recycling system and have a complex relationship with each other. To analyze the relationship among key stakeholders more effectively, this study conducted interviews (telephonic or face to face) with randomly selected representatives of Internet recycling companies, traditional recyclers, residents, and the government. The main contents of the interview survey are shown in Table 8.

#### 3.3.1. Relationship among Key Stakeholders in the Recycling of Waste Household Appliances

**Figure 2:**
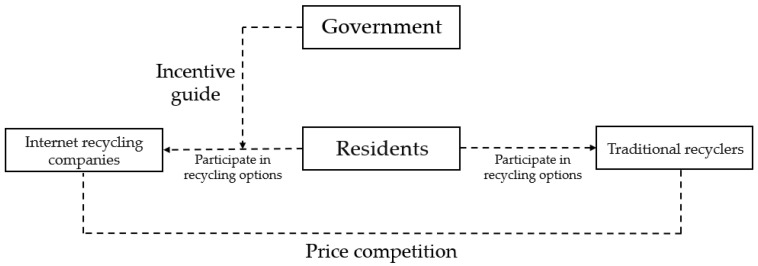
Relationships between stakeholders in the recycling of household appliance waste.

Due to the large size of TV sets, refrigerators, and other household appliances, the proportion of residents who leave them at home or directly discard them is small. Therefore, the situation that residents do not participate in recycling is no longer considered, and only traditional recycling or Internet recycling is considered. Because the operating cost of Internet recycling companies is higher than that of traditional recycling companies, traditional recycling companies have a competitive advantage in the recycling price. To promote residents’ participation in Internet recycling, the government needs to provide incentives and guiding measures. Figure 2 shows the relationship between stakeholders in the recycling of household appliance waste.

In the recycling of household appliance waste, price competition exists between Internet recycling companies and traditional recycling companies. The recycling price is an important factor for residents to consider when choosing to participate in recycling. In consideration of the development of the WEEE Internet recycling industry, the government will need to enhance residents’ participation in Internet recycling, so residents should be encouraged or guided to participate therein.

#### 3.3.2. Relationship among Key Stakeholders in the Recycling of Waste Electronic Products

**Figure 3:**
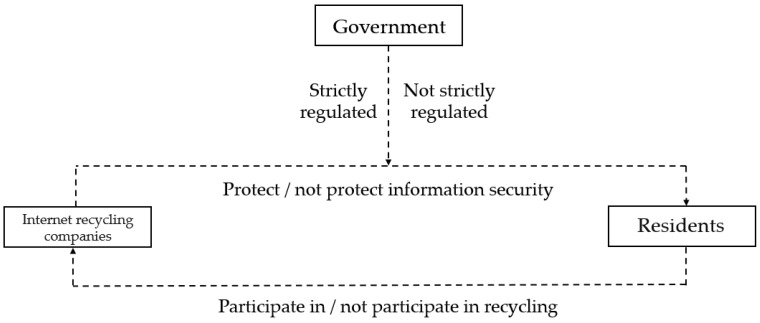
Relationships between stakeholders in the recycling of WEEE.

For the recycling of waste electronic products, residents are more concerned about information security. Compared with traditional recyclers, Internet recycling companies are more disorganized; hence, the government cannot effectively supervise them, and information security cannot be guaranteed. Therefore, the government, Internet recycling companies, and residents are regarded as stakeholders in the recycling of electronic product waste. The relationship between stakeholders in the recycling of electronic product waste is shown in Figure 3.

Due to the particularity of WEEE, although the recycling price is relatively high, residents opt for idle disposal over recycling for fear that their information security cannot be guaranteed. When recycling electronic products, internet recycling companies promise to crush residents’ information to ensure that the information will not be leaked. However, more technical costs need to be invested to protect information security. If this cost will make it difficult for companies to make profits, their commitment to information security protection may not be realized. Therefore, government supervision plays an important role. Under the strict supervision of the government, whether Internet recycling companies really protect information security will be disclosed, and the information will be more transparent. Residents’ fear of information leakage will be reduced, and residents’ participation in Internet recycling will be enhanced.

## 4. Discussion

Through the identification and division of stakeholders, this study identifies the government, residents, Internet recycling companies, and traditional recyclers as key stakeholders in the WEEE Internet recycling industry. In the management of WEEE, the key stakeholders all play a vital role in the development of the industry and form the core part of the industry stakeholders. In the evolutionary game analysis of WEEE recycling stakeholders by Wang et al. [24], the government, residents, and recyclers were also regarded as the three key stakeholders. From the WEEE management policies of various countries, we can also find that for the management of the WEEE recycling industry, most countries have implemented plans for different stakeholders such as recyclers, manufacturers, and consumers [25]. This shows that this view is also mainly applied in the management practice of the WEEE recycling industry.

In addition, we found that Internet recycling companies are also key stakeholders in the industry, ranking first in terms of initiative and importance, and second in terms of urgency. The role played by Internet recycling companies in the industry deserves our attention. Moreover, the current Internet recycling industry has not been fully developed, and Internet recycling will play a greater role in the near future. Some studies have begun to focus on the field of Internet recycling and have demonstrated its effectiveness [26,27]. However, the potential for Internet recycling deserves more attention, a more systematic network, and a better assessment.

In the research, we found some marginal stakeholders. They are passively affected by the development of Internet recycling and have the lowest impacts on the development of Internet recycling. Therefore, it is easier to ignore them in our management. But these stakeholders are actually very important and cannot be ignored. Hazardous waste treatment companies are one example. Due to the polluting nature of WEEE, if the industry lacks this stakeholder, an adverse impact of WEEE on the environment will be unavoidable. They are classified as marginal stakeholders, which only means that they contribute less to the development of the industry, not that they can be ignored. Instead, we should also pay attention to them.

Finally, during our interviews, we discovered a phenomenon worth exploring. An important reason for the high idle rate of waste electronic products is the information security of consumers. Consumers often change their recycling behavior because they fear information leakage. This was confirmed in a study by Zhang et al. [28]., who found that privacy issues have a direct positive impact on the willingness to formally recycle, with people tending to be more willing to leave their used mobile phones at home, while those who are willing to participate in recycling prefer formal recycling channels. This problem is worthy of our attention and is a problem that must be solved in the process of establishing a perfect recycling network.

Overall, the most important contribution of this work to other research is to identify stakeholders within the WEEE Internet recycling industry and compare their importance to find key stakeholders. This can help relevant studies to select research objects and design research directions when conducting stakeholder analysis, at the same time as finding gaps in research.

## 5. Conclusions and Policy Implications

### 5.1. Conclusions

The Mitchell scoring method was used to identify the key stakeholders of WEEE Internet recycling and to analyze their behavioral characteristics and relationships. The results show that the government, Internet recycling companies, traditional recyclers, and residents are the key stakeholders of WEEE Internet recycling. The behaviors of different key stakeholders interacted with each other, thus affecting the development of WEEE Internet recycling. Finally, the interrelationship between key stakeholders was different in the field of electrical appliance waste recycling and electronic product waste recycling. In the recycling of waste electronic products, residents choose whether to participate based on comprehensive recovery income and information security. Internet recycling companies need to invest in the protection of residents’ information security. To encourage residents to participate in recycling, the government needs to supervise Internet recycling companies.

### 5.2. Policy Implications

#### 5.2.1. Develop an Emerging Recycling Mode

In the management of the WEEE recycling industry, we should vigorously develop the emerging recycling mode (e.g., “Internet +” recycling method) and provide Internet companies with more convenient conditions, perfect systems, and reasonable and fair standards, which can further enhance the important role they play in the industry. To improve the efficiency and convenience of WEEE recycling, China’s WEEE recycling industry needs a more advanced recycling system.

#### 5.2.2. Cultivate Residents’ Environmental Awareness

Improving residents’ awareness of environmental protection and establishing good environmental protection habits can greatly improve the recycling efficiency of WEEE, and improve the garbage sorting and recycling mechanisms, to further leverage the stakeholders who are restricted by the environment and cannot play a role, such as garbage treatment companies.

#### 5.2.3. Strengthen Information Security Management

One of the serious problems that lead to the idleness of WEEE is that information security is difficult to guarantee. Improving the management of information security is not only an important problem to be solved urgently in building an Internet recycling network, but also an important method for encouraging consumers to participate in the recycling industry with peace of mind. Hence, these aspects should be focused on to manage WEEE in a safe and efficient manner.

## Figures and Tables

**Table 1:** Global stakeholder study sheet on Internet recycling of electronic waste.

Research Object	Stakeholders	Ref *
WEEE network recycling system	Network recycling platform operators, consumers, recyclers, formal environmental protection processing companies, logistics companies, third-party payment, network technology service providers, governments, charitable and public welfare organizations, media, scientific research institutions, other cooperative companies, industry associations, competitive recyclers, manufacturers, and the public	[16]
Waste electrical appliances recycling	Manufacturers, retailers, consumers, processors, governments, third party organizations	[17]
The urban mining	Residents, production companies, recycling companies, mobile recycling vendors, scavengers, waste disposal companies, waste treatment plants, individual maintenance stations, and governmental and non-government organizations	[18]
O2O recycling service system for renewable resources	Community residents, recycling personnel, renewable resources recycling platform, recycling platform staff, manufacturers, property and environmental protection personnel, communities, industrial and commercial departments, sanitation departments	[19]
German WEEE treatment system	Ministry of Environment, coordination center, consumers, recycling point, manufacturers, distributors, processors and logistics transporters	[20]
Intervention measures on recycling of WEEE	Consumers, recyclers, collectors, government	[21]
WEEE social recycling behavior	Manufacturers, consumers, recyclers and disposers, government	[22]

* Ref, references.

**Table 2:** WEEE internet recycling stakeholders.

	The Research Object	Stakeholders
1	Internet recycling companies	The main recyclers of the WEEE Internet recycling industry, adopting a combination of online and offline ways to recycle WEEE
2	Government	The main regulator of the industry, formulating policies to supervise and constrain other stakeholders
3	Residents	The main producers of WEEE, directly involved in the Internet recycling of WEEE
4	Traditional recyclers	Individual stations and traditional recycling companies competing with Internet recycling companies
5	Electronic product manufacturers	Producers of electronic products and participants in the recovery and treatment of WEEE
6	Disassembly and treatment companies	Companies downstream of the Internet recycling industry that play a role in the dismantling and processing of WEEE
7	Reusing companies	Companies that recycle and produce renewable resources obtained from the disassembled WEEE
8	Hazardous waste treatment companies	Companies that handle the treatment of hazardous waste generated from disassembled WEEE
9	Non-governmental organizations	Regulators other than the government
10	Garbage treatment companies	Companies involved in the final treatment of waste that cannot be reused after disassembly

**Table 3:** WEEE internet recycling industry stakeholder classifications.

Dimension	Initiative			Importance			Urgency		
Types	[1–3]	(3–4]	(4–5]	[1–3]	(3–4]	(4–5]	[1–3]	(3–4]	(4–5]
Key stakeholders	-	-	√	-	-	√	-	-	√
	-	-	√	-	-	√	-	-	-
	-	-	√	-	-	-	-	-	√
	-	-	-	-	-	√	-	-	√
Potential stakeholders	-	√	-	-	√	-	-	√	-
	-	√	-	-	√	-	-	-	-
	-	√	-	-	-	-	-	√	-
	-	-	-	-	√	-	-	√	-
Marginal stakeholders	√	-	-	√	-	-	√	-	-
	√	-	-	√	-	-	-	-	-
	√	-	-	-	-	-	√	-	-
	-	-	-	√	-	-	√	-	-

**Table 4:** WEEE internet recycling industry stakeholder initiative scores.

Stakeholders	Number	Minimum	Maximum	Mean	Rank
Internet recycling companies	16	4	5	4.88	1
Governments	16	3	5	3.81	2
Residents	16	2	5	3.56	4
Traditional recyclers	16	3	5	3.69	3
Electronic product manufacturers	16	1	4	2.69	7
Disassembly and treatment companies	16	2	4	2.94	6
Reusing companies	16	1	4	3.06	5
Hazardous waste treatment companies	16	1	4	2.06	9
Non-governmental organizations	16	1	4	2.56	8
Garbage treatment companies	16	1	3	1.88	10

**Table 5:** WEEE internet recycling industry stakeholder importance scores.

Stakeholders	Number	Minimum	Maximum	Mean	Rank
Internet recycling companies	16	5	5	5.00	1
Governments	16	3	5	4.50	2
Residents	16	3	5	4.17	3
Traditional recyclers	16	3	5	4.06	4
Electronic product Manufacturers	16	1	5	3.44	5
Disassembly and treatment companies	16	2	4	3.17	6
Reusing companies	16	2	4	3.11	7
Hazardous waste treatment companies	16	1	4	2.11	7
Non-governmental organizations	16	1	4	2.28	9
Garbage treatment companies	16	1	4	2.11	10

**Table 6:** WEEE internet recycling industry stakeholder urgency scores.

Stakeholders	Number	Minimum	Maximum	Mean	Rank
Internet recycling companies	16	2	5	4.11	2
Governments	16	3	5	4.39	1
Residents	16	3	5	4.11	2
Traditional recyclers	16	3	5	4.06	4
Electronic product manufacturers	16	2	4	3.11	5
Disassembly and treatment companies	16	2	4	3.06	6
Reusing companies	16	2	4	2.89	7
Hazardous waste treatment companies	16	1	3	2.00	9
Non-governmental organizations	16	1	4	2.56	8
Garbage treatment companies	16	1	3	1.94	10

**Table 7:** Three-dimensional classification results of WEEE Internet recycling stakeholders.

	[1–3]	(3–4]	(4–5]
Initiative	Electronic product manufacturers, disassembly and treatment companies, hazardous waste treatment companies, non-governmental organizations, garbage treatment companies	Government, residents, traditional recyclers, reusing companies	Internet recycling companies
Importance	Hazardous waste treatment companies, non-governmental organizations, garbage treatment companies	Electronic product manufacturers, disassembly and treatment companies, reusing companies	Internet recycling companies, government, residents, traditional recyclers
Urgency	Reusing companies, hazardous waste treatment companies, non-governmental organizations, garbage treatment companies	Electronic product manufacturers, disassembly and treatment companies	Internet recycling companies, government, residents, traditional recyclers

**Table 8:** Interview information.

Respondents	Number of Respondents	The Interview Form	Main Contents of Interview
Internet recycling companies	3	Telephonic, Face-to-face	What do you think are the factors that affect the Internet recycling of household appliances and electronic product waste? What are the influences of traditional recyclers, government, and residents on your development?
Traditional recyclers	2	Telephonic	What do you think are the factors that affect the Internet recycling of household appliances and electronic product waste? What impact do Internet recycling companies, governments, and residents have on your development?
Residents	5	Face-to-face	Do you prefer traditional recycling or Internet recycling when recycling electrical appliances and electronic product waste? What’s the reason?
Governments	1	Telephonic	What policies do you think are needed for both Internet and traditional WEEE recycling? What incentives do residents need?

## Data Availability

The data investigated during this study are available from the corresponding author upon reasonable request.
